# Bioactive Molecule from *Streptomyces* sp. Mitigates MDR *Klebsiella pneumoniae* in Zebrafish Infection Model

**DOI:** 10.3389/fmicb.2017.00614

**Published:** 2017-04-12

**Authors:** Lalitha Cheepurupalli, Thiagarajan Raman, Sudarshan S. Rathore, Jayapradha Ramakrishnan

**Affiliations:** ^1^Actinomycetes Bioprospecting Lab, Centre for Research in Infectious Diseases, School of Chemical and Biotechnology, SASTRA UniversityTirumalaisamudram, India; ^2^Laboratory for Inflammation Research, Centre for Research in Infectious Diseases, School of Chemical and Biotechnology, SASTRA UniversityTirumalaisamudram, India

**Keywords:** carbapenem resistant, MDR *Klebsiella pneumoniae*, zebrafish, *Streptomyces* sp. ASK2, bioactive compound

## Abstract

The emergence and spread of multi-drug resistant (MDR) especially carbapenem-resistant *Klebsiella pneumoniae* is a major emerging threat to public health, leading to excess in mortality rate as high as 50–86%. MDR *K. pneumoniae* manifests all broad mechanisms of drug resistance, hence development of new drugs to treat MDR *K. pneumoniae* infection has become a more relevant question in the scientific community. In the present study a potential *Streptomyces* sp. ASK2 was isolated from rhizosphere soil of medicinal plant. The multistep HPLC purification identified the active principle exhibiting antagonistic activity against MDR *K. pneumoniae*. The purified compound was found to be an aromatic compound with aliphatic side chain molecule having a molecular weight of 444.43 Da. FT-IR showed the presence of OH and C=O as functional groups. The bioactive compound was further evaluated for drug induced toxicity and efficacy in adult zebrafish infection model. As this is the first study on *K. pneumoniae* – zebrafish model, the infectious doses to manifest sub-clinical and clinical infection were optimized. Furthermore, the virulence of *K. pneumoniae* in planktonic and biofilm state was studied in zebrafish. The MTT assay of *ex vivo* culture of zebrafish liver reveals non-toxic nature of the proposed ASK2 compound at an effective dose. Moreover, significant increase in survival rate of infected zebrafish suggests that ASK2 compound from a new strain of *Streptomyces* sp. was potent in mitigating MDR *K. pneumoniae* infection.

## Introduction

The emergence and spread of pathogens harboring extended spectrum β-lactamase-like carbapenem-resistant *Klebsiella pneumoniae* (CR-KP), *Escherichia coli*, and other gram negative bacteria are major emerging threat to public health (Schwaber and Carmeli, 2008; Vatopoulos, 2008). Of particular concern is the spread of multi-drug resistant (MDR) strains of *K. pneumoniae*, an encapsulated opportunistic pathogen, colonizes the human gastrointestinal tract, skin, nasopharynx and urinary tract. The pathogen is characterized by the presence of major virulence factors such as capsule (Siu et al., 2012), type 1 and type 3 pili (Di Martino et al., 2003), LPS (Vuotto et al., 2014) and siderophores (Schembri et al., 2005). Among which, the thick polysaccharide capsule is a promising virulence factor in *K. pneumoniae* that facilitate its evasion of host defenses (Yu et al., 2008). During infectious state, *K. pneumoniae* grow as a biofilm on medical equipments like catheters and this is not only crucial for the establishment of infection but also makes treatment difficult (Zowawi et al., 2015). *K. pneumoniae* is the major cause of infections in catheterized patients, and hence categorized as one of the top eight significant nosocomial pathogens (Ullmann, 1998; Khan et al., 2015). Added to all, *K. pneumoniae* is emerged as a new MDR varieties of human pathogens that can have drastic consequences on health care worldwide (Patel et al., 2008; Struelens et al., 2010; Ulu et al., 2015). One such best example is the evolution and spread of CR-KP. Extended spectrum β-lactamase (ESBL) and carbapenamase producing *K. pneumoniae* have been shown to manifest all broad mechanisms of drug resistance (Nordmann et al., 2009) and also confer resistance to other class of antibiotics such as aminoglycosides, sulphonamides, trimethoprim, tetracyclines, and chloramphenicol, as the plasmids coding for ESBLs may also carry additional genes conferring resistance to many other antimicrobial classes (Rawat and Nair, 2010). Various drug resistance mechanisms including gene mutation, acquisition of novel antibiotic catalytic genes, modification of target site and membrane proteins, differential expression of specific genes for efflux pumps which mediate drug effects and biofilm formation (Tenover, 2006; Nordmann et al., 2009; Cooksey, 2011). Hence infections caused by CR-KP are hard to treat (Kumar et al., 2011; Thakur et al., 2013). Currently, clinicians follow one of the three strategies for the treatment of CR-KP, the first line antibiotic such as meropenem, fluoroquinolone, and aminoglycoside. However, at higher concentrations, these drugs are highly toxic. The second choices of drugs are colistin, tigecycline, and fosfomycin, are more toxic than the fist-line drugs (Melano et al., 2003; Petrosillo et al., 2013).

Unfortunately, the emergence of colistin resistance limits further treatment options leading to increase in mortality rates to as high as 50–86% (Hirsch and Tam, 2010; Tzouvelekis et al., 2012; Capone et al., 2013). The third approach, combination therapy has been shown to be a promising choice to overcome the emergence of drug resistance and minimizing drug toxic effect, but in terms of outcomes there are very few reports illustrating combination therapy in humans (Kontopidou et al., 2014). Hence to combat the occurrence of resistant bacteria, development of new antimicrobials has become a more relevant question in the scientific community.

Rhizosphere soil of medicinal plants constitutes one such source for exploring antimicrobials owing primarily to their rich biodiversity (Khamna et al., 2009). *Streptomyces* spp. are one such group of microbes with huge and untapped potential for antimicrobial production. Thus many scientific communities are exploring actinomycetes spp. from various habitats for the development of novel anti-infectives. Rhizosphere soil are rich source of microbes, as rhizodeposition provides an important substrate for soil microbial community (Haichar et al., 2008), especially medicinal plants due to their unique and enormously divergent bioactive molecules. Hence, we have selected few medicinal plants to isolate actinomycetes spp. antagonistic against MDR *K. pneumoniae.*

During early stage of drug development, drug validation is an important step. Zebrafish (*Danio rerio*) are widely accepted model in the current decade for *in vivo* assessment of drug efficacy (Kari et al., 2007). The major advantages of zebrafish as a predictive model for assessing drug induced toxicity includes the similarity of toxicity profiles to that of humans, requirement of small amount of drug, simple route of administration and also the efficacy of drug can be studied for different infectious dose (Li and Hu, 2012).

In the present study, we have selected five different medicinal plants to isolate potential *Streptomyces* sp. antagonistic against MDR *K. pneumoniae.* The bioactive molecule was isolated by multistep HPLC purification. The toxicity and efficacy of the bioactive molecule were evaluated using adult zebrafish. Also we optimized infectious dose to manifest sub-clinical and clinical infections using planktonic and biofilm of *K. pneumoniae*. Even though many zebrafish infectious models have been developed, this is the first study to report *K. pneumoniae* – zebrafish model and *ex vivo* organ culture of zebrafish liver to screen drug induced toxicity.

## Materials and Methods

### Screening of Multi-drug Resistance in *K. pneumoniae*

Multi-drug resistant *K. pneumoniae* was obtained from Department of Microbiology, Aravind Eye Hospital, Madurai, India. The presumptive identification of the isolate was done using HiCrome UTI agar (HiMedia, India). Further identification were made by performing biochemical tests such as Indole production, MR-VP test, urease test, and lactose fermentation at 44.5°C for 5 days (Ullmann, 1998; Podschun et al., 2001).

Further, the strain was screened for drug resistance by performing Kirby-Bauer disk diffusion test against various selected antibiotics according to Clinical Laboratory Standards Institute (CLSI) guidelines. The selected antibiotics cover a range of β-lactams, fluoroquinolones, aminoglycosides, tetracyclines and polypeptides. Commercially available antibiotics disks (HiMedia, India) were used. The details of antibiotics used in the present study are given in **Table 1**. Triplates were maintained and the results were recorded as sensitive or resistant according to CLSI standard chart.

**Table 1:** Antimicrobial susceptibility pattern of clinical strain of *K. pneumoniae.*

Antibiotic name	Quantity of antibiotic	Resistance/sensitive/intermediate
**β-lactums**		
**I. Penicillins**		
Oxacillin	1 μg	Resistance
Methicillin	5 μg	Resistance
**II. Cephalosporins**		
**a. First generation antibiotics**		
Cefazolin	30 μg	Resistance
Cefadroxil	30 μg	Resistance
Cefalexin	30 μg	Resistance
**b. Second generation antibiotics**		
Cefuroxime	30 μg	Resistance
**c. Third generation antibiotics**		
Cefotaxime	30 μg	Resistance
Ceftazidime	30 μg	Resistance
Ceftazoxime	30 μg	Resistance
**d. Fourth generation antibiotics**		
Cefepime	30 μg	Resistance
Cefazolin	30 μg	Resistance
**III. Carbapenems**		
Imipenem	10 μg	Resistance
Meropenem	10 μg	Resistance
Ertapenem	10 μg	Resistance
Doripenem	10 μg	Resistance
**IV. Aminopenicillins**		
Amoxicillin	10 μg	Resistance
Ampicillin	10 μg	Resistance
**Fluoroquinolones**		
Levofloxacin	5 μg	Resistance
Gatifloxacin	5 μg	Resistance
Moxifloxacin	5 μg	Resistance
Ofloxacin	5 μg	Resistance
**Aminoglycosides**		
Gentamicin	30 μg	Resistance
Tobramycin	10 μg	Resistance
Amikacin	10 μg	Sensitive
**Tetracyclines**		
Tetracycline	30 μg	Resistance
**Polypeptides**		
Polymyxin B	300 units	Sensitive

### Screening of ESBL Production

Double disk synergy method was performed to detect ESBL production as recommended by CLSI (2011b). The method was executed using cefotaxime (30 μg) alone and in combination with clavulanic acid (10 μg). The cefotaxime disks were placed 30 mm apart from clavulanic–cefotaxime disk. The increase in zone toward the disk of cefotaxime–clavulanate was considered as positive for ESBL production (CLSI, 2011a; Kumar et al., 2014).

### Rhizosphere Soil Collection and Pretreatment

Rhizosphere soil of five different medicinal plants (*Solanum trilobatum, Ocimum tenuiflorum, Cardiospermum halicacabum, Justicia adhatoda*, and *Wedelia chinensis*) were collected from local medicinal plant garden. The plants were removed with intact roots and root zone soil were collected in a sterile container and kept at 4°C for further processing (Turnbull et al., 2012). Ten gram of each soil samples was incubated at 60°C for 40 min and re-suspended in 50 ml of saline. The re-suspended mixture was diluted with 50 ml of saline containing 1.5% (v/v) phenol and shaken for 30 min at 28°C (Ramakrishnan et al., 2009).

### Isolation of Actinomycetes spp.

The actinomycetes spp. were isolated by serially diluting the pretreated rhizosphere soil sample and inoculating by spread plate method onto various agar medium recommended for Actinomycetes spp. such as glycerol asparagine agar, actinomycetes isolation agar (Nanjwade et al., 2010), tryptone yeast extract medium (ISP1), yeast extract malt extract agar (ISP2) (Busarakam et al., 2014) and Czapek dox agar (Mangamuri et al., 2016). The media were supplemented with gentamicin (1 μg/ml) and fluconazole (50 μg/ml) after sterilization to inhibit the growth of bacteria and fungi, respectively (Eccleston et al., 2008). The plates were then incubated for 21 days at 30°C. All the morphologically different actinomycetes sp. grown on different agar medium were sub-cultured. Further, 20% of glycerol stocks at -80°C were maintained.

### Antagonistic Activity against MDR *K. pneumoniae*

Anti-bacterial activity of 51 actinomycetes isolates were assayed to select a potential strain according to Ramakrishnan et al. (2009). Spore suspensions of individual isolates were spot inoculated (10 μl per spot) on Muller Hinton agar plates, and incubated at 30°C for 3 days. The cells were then killed with chloroform vapors and were subsequently over laid with 15 ml of medium containing 1% (w/v) agar, 0.5% (w/v) peptone, 0.5% (w/v) yeast extract and inoculated with 100 μl of test isolate MDR *K. pneumoniae* on the agar surface. The resulting clear zone of inhibition (ZOI) was measured after 24 h of incubation. The experiment was repeated thrice. Mean diameter of ZOI and standard deviations were calculated. The strain ASK2 which exhibited the antagonistic activity against MDR *K. pneumoniae* was selected for taxonomical investigation.

### Taxonomical Investigation of ASK2

Genomic DNA from *Streptomyces* sp. ASK2 was isolated using the procedure described by Kimura (1980). The gene fragments were amplified by using PCR Kit (GENEI Pvt. Ltd, India) and the 16S rRNA gene was amplified using Mastercycler pro thermal cycler (Eppendorf) with the following profile: initial denaturation at 95°C for 4 min, 30 amplification cycles of (95°C for 1 min, annealing temperature at 50°C for 60 s, 72°C for 1 min) and a final extension step at 72°C for 4 min. The PCR product was electrophoresed and purified from 1.5% agarose gel using QIAquick PCR purification kit (QIAGEN) and sequenced using the primers 8F and U1492R (Abdel Azeiz et al., 2016). Sequencing was done at Chromous Biotech, Bengaluru, India using ABI 3100 sequencer (Applied Biosystems). The sequence was edited using FinchTV (Geospiza, Inc.) and BioEdit (Ibis Biosciences, Abbott Labs). Sequence similarity search was made using 16S rRNA gene and taxid specific BLAST tool. Representative 16S rRNA sequences of related type strains of *Streptomyces* sp. were retrieved from National Center for Biotechnology Information (NCBI) database, and were aligned with *Streptomyces* sp. ASK2 using CLUSTAL-X software (Ser et al., 2015a). MEGA version 5.2.2 neighbor-joining algorithm used to construct phylogenetic tree (Tan et al., 2015). Kimura’s two-parameter model was used to compute evolutionary distances for the neighbor-joining algorithm (Ser et al., 2015b).

### Fermentation and Recovery of Biomolecules

The seed culture of ASK2 was grown on seed medium (1% starch, 0.5% glucose, 0.5% yeast extract, 0.5% K_2_HPO_4_, 0.05% MgSO_4_7H_2_0) and incubated at 30°C for 3 days on a rotary shaker. 15% (v/v) of seed culture was used to seed 10 liters of production medium (1% starch, 0.5% glucose, 0.5% yeast extract, 0.5% K_2_HPO_4_, 0.05% MgSO_4_ and 0.5% peptone) at 30°C for 11 days. The fermented broth was centrifuged at 4300 × *g* for 10 min at 4°C. The culture filtrate was extracted with equal volume of ethyl acetate (EtOAc) and concentrated using rotary vacuum evaporator. The EtOAc extract was further concentrated *in vacuo* and dissolved in 10X phosphate buffer saline pH 7.0.

### Purification

The EtoAc extract dissolved in phosphate buffer was purified using Semi-preparatory HPLC (Agilent Technologies, India). Reverse-phase analytical C18 column (Agilent Technologies, Netherlands: 4.6 mm × 250 mm) was used for standardizing the experimental conditions. The chromatographic separation was carried out using C18 preparatory column (Agilent Technologies, Netherlands: 10 mm × 250 mm) with water and methanol, 60:40, flow rate of 4 ml/min and injection volume of 0.5 ml. The elution pattern was monitored at 250 nm, peaks were then eluted out separately and each of them was screened for bioactivity by means of broth dilution method. The broth system consisted of 30 μl of 0.5 McFarland of *K. pneumoniae* culture in 2 ml nutrient broth with 200 μl of purified compound. The similar protocol was followed to find the activity against the standard isolate obtained from Microbial culture collection, Pune, MCC 2570-*Klebsiella pneumoniae*, NDM type drug resistant strain.

The fraction which displayed antimicrobial activity was further processed for second and third step purification by using water and methanol (40:60) as a mobile phase.

### Characterization

#### Fourier Transform–infra-red Spectroscopy

The HPLC purified ASK2 was studied by Fourier transform–infra-red spectroscopy and the characteristics bands between 4000 and 400 cm^-1^ were recorded using Perkin Elmer, Spectrum RX I.

#### Mass Spectroscopy Analysis

The purified compound was subjected to Electron Spray Ionization and the molecular weight was determined from m/z values.

#### ^1^H-NMR

The purified ASK2 was dissolved in DMSO and spectral analysis was performed using BRUCKER 300MHz AVANCE-II.

### MIC Determination

The MIC of the compound ASK2 against *K. pneumoniae* was determined for the active fraction using microbroth dilution assay as described in the CLSI protocols. The overnight *K. pneumoniae* culture was diluted to obtain the final concentration of 10^5^ cfu/ml. The compound was dissolved in PBS and was tested at concentrations from 0.5 to 64 μg in twofold step intervals. Each well contains 100 μl of nutrient broth, 10 μl of culture and 100 μl of compound with varying concentrations. Triplicates were maintained for each concentration. The OD values were recorded at 600 nm, as well, the colony counts were recorded on Nutrient agar plates. The OD values and colony forming units were used to determine the MIC.

### Animals

Zebrafish (*D. rerio*) irrespective of sex measuring approximately 4 to 5 cm in length, and weighing 300 mg, were purchased from a local aquarium. Proper care and maintenance such as feed, aeration and 14:10-h light/dark cycle at 25°C were ensured to allow acclimatization for a week before the study. All experiments were performed in compliance with applicable national and/or institutional guidelines for the care and use of animals (Animal Biosafety Level 2) were followed.

### Preparation of Different Concentration of *K. pneumoniae*

*Klebsiella pneumoniae* were grown overnight in nutrient broth at 37°C. Different concentrations of cells were prepared by observing optical density and by plating on solid media ranging from 10^4^ to 10^14^ cfu/ml.

### Biofilm Formation

A loopful of *K. pneumoniae* cells were inoculated in 50 ml nutrient broth and incubated at 37°C for 18 h. The cells were collected by centrifugation and adjust to 0.5 McFarland (10^8^ cfu/ml) with nutrient broth. 500 μl of inoculum were added to wells of 6 well cell culture plates containing glass coverslip of 1 cm^2^ and incubated at 37°C for 72 h. The media were discarded and non-adherent cells were removed by washing with PBS. The biofilms that remained over the coverslips were scrapped and suspended in PBS and optical density of biofilm was measured at OD_600._

### Induction of *K. pneumoniae* Planktonic and Biofilm Infection

To establish *K. pneumoniae* infection in zebrafish, planktonic cells of different density (10^4^–10^14^ cfu/ml) and biofilm (10^9^–10^14^ cfu/ml) were used. Each test group (*n* = 5 for each group) were infected by injecting 10 μl of different density of planktonic cells of intramuscularly 45° angle to the spine into a position immediately lateral to the Dorsal fin at a depth of 2 mm using sterile 1 ml insulin syringe (U-40, 0.30 mm × 8 mm). One uninfected group was maintained as control. The fish were observed daily to monitor pathological changes such as infection and lethality. The fish which were alive were sacrificed after 24 h and the dead fish were dissected immediately (∼6 hpi). The selected infected and uninfected whole fish were placed on iced wax plate, the organs were dissected out and the muscle tissue was used to estimate *K. pneumoniae* loads in. 100 mg of dissected muscle tissue were placed in a 2 ml microcentrifuge tube and homogenized using micropestle in 0.5 ml phosphate buffer saline and made upto 1 ml. This was followed by serial dilutions of 10^-1^ to 10^-5^ homogenates using sterile PBS and counts of blue mucoid colonies were estimated on UTI agar medium using 10^-5^ dilution. The colonies were counted following 24 h incubation at 37°C.

### Toxicity Assessment

The compound was dissolved in phosphate buffer (10X) and fixed dose procedure was followed to assess the non-lethal toxicity. The following concentrations of compound were evaluated (8, 16, 32, 64, and 128 μg/ml). Zebrafish were challenged with intramuscular injection of 10 μl of different doses of compound (*n* = 5), and control groups received phosphate buffer as vehicle control (*n* = 5). Zebrafish were monitored for behavioral changes and mortality for upto 120 h. The experiments were repeated thrice on three independent days.

### *Ex Vivo* Culture of Liver and Cell Viability Assay

**FIGURE 1:**
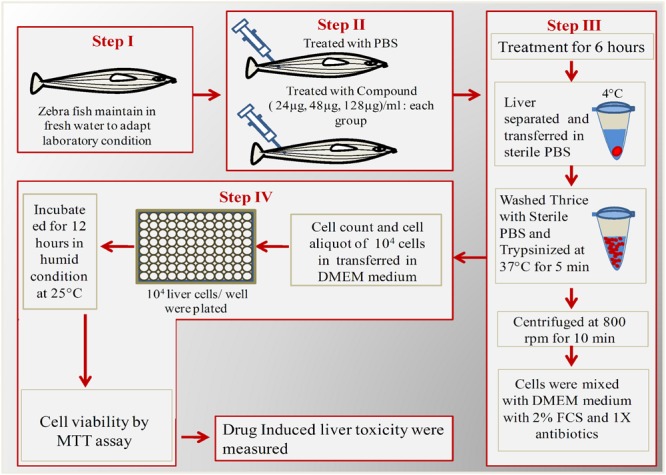
**Schematic overview of *ex vivo* organ culture of zebrafish liver cells.** Step I: Acclimatization of zebrafish for 1 week; Step II: Injected with different concentrations of ASK2 and observe for 6 h; Step II: Separation and trypsinization of liver; Step IV: Plating of liver cells in 96 well plate for over night and checking cell viability by MTT assay.

Liver toxicity assay were performed for low dose (24 μg/ml), effective dose (48 μg/ml), and high dose (128 μg/ml) of ASK2. Effective dose is the one that was sufficient to achieve the desired clinical improvement (survival) after being challenged with infectious dose of *K. pneumoniae* cells. The toxicity was evaluated by MTT cell viability assay in *ex vivo* organ culture of zebrafish liver. The protocol developed for hepatotoxicity assessment in *ex vivo* organ culture of zebrafish is provided in **Figure 1**.

Each test group (*n* = 5) was administered with 24, 48, and 128 μg/ml of drug concentrations, and the control group received PBS. After 12 h, the fish were anesthetized by 150 mM of tricaine-S (MS-222) and euthanized by decapitation, the liver was isolated and washed thrice with PBS (experiment was carried at 4°C). The cleaned and chilled liver was trypsinized for 5 min to obtain single cells. To allow cell adherence, trypsin was then neutralized with culture media (DMEM + 10% FCS + 1X antibiotics). The dissociated cells were centrifuged (43 × *g* for 5 min) at 4°C and the cell pellets were re-suspended in growth media. The cells were seeded in a 96 well flat bottom microtitre plate at a density of 10^4^ cells/ml with 100 μl of culture medium. A control well (culture medium alone) was maintained. The plate was incubated at 37°C for 12 h to allow the liver cells to grow and adhere. After incubation, the medium from the wells were removed carefully and MTT assay was performed. Briefly, 10 μl of MTT solution was added and the plates were incubated for 4 h at 28°C. At the end of the incubation, the culture medium was removed and 100 μl of DMSO was added and allowed to react for 45 s. The readings were obtained at 570 nm using ELISA plate reader. Meanwhile, the viability of cells were also assessed using fluorescent microscopy using FITC and propidium iodide stain to discriminate live and dead cells. After the removal of cell culture medium, the staining solution was added and allowed to stain for 15 min in dark. The staining solution was removed by washing with PBS and the samples were analyzed using Nikon Trinocular microscope (Nikon Eclipse Ni-U, Japan).

### Histopathology

Selected whole zebrafish were fixed following wash with 0.9% ice-cold saline and fixed immediately by immersion in Dietrich’s fixative (10 ml per fish), with overnight incubation at room temperature. Fixed samples were routinely processed and then embedded in paraffin; 5 mM thick longitudinal sections were prepared which were de-waxed and rehydrated by standard methods and stained by haematoxylin and eosin. The histological observation of muscle tissue were made using Nikon Trinocular microscope (Nikon Eclipse Ni-U, Japan).

### Efficacy of Compound to Treat MDR *K. pneumoniae* Infection

Two groups of fish were infected with 10 μl of 10^12^ cells intramuscularly. After 3 h of infection, the treatment group (*n* = 20) were intramuscularly administered with effective dose (48 μg/10 μl) of ASK2. The control group received sterile PBS as vehicle control. All the groups were monitored for the behavioral changes and mortality for 120 h. The survival rate and *K. pneumoniae* burden were recorded.

### Statistical Analysis

All experiments were performed in triplicates. Mean, Standard deviation and Student’s *t*-test was performed to test the statistical significance in MTT assay studies and graphs were prepared by using Graphpad prism 6.

## Results

### Screening of Multi-drug Resistance in *K. pneumoniae*

Presumptive identification of *K. pneumoniae* using chromogenic agar results in the formation of blue mucoid colonies (Rajaratnam et al., 2014). Further, the use of conventional phenotypic methods allowed the characterization of *K. pneumoniae* as mucoid, non-motile, encapsulated isolate exhibiting positive reactions for VP test, urease test, lactose fermentation at 45°C and Indole negative, which basically distinguish it from *K. oxytoca* (Brisse et al., 2006; Patel et al., 2008). The antimicrobial susceptibility of *K. pneumoniae* against various antibiotics are given in **Table 1**. It was observed that the clinical *K. pneumoniae* exhibits resistance to all antibiotics of different classes including β-lactams, fluoroquinolones, aminoglycosides, tetracyclines and polypeptides and shows sensitivity to polymyxins alone. Further, the test strain shows positive for ESBL screening using cefotaxime–clavulanate combined disk test as recommended by CLSI. The test strain showed 25 mm of ZOI for cefotaxime-clavulanate combined disk (30/10 μg) whereas absence of ZOI was observed for cefotaxime disk. This result confirms that the clinical strain *K. pneumoniae* used in the present study produces ESBL. Hence the clinical strain was designated as MDR bacteria as per ECDC and CDC standard definition (Magiorakos et al., 2011).

### Anti CR-KP Activity and Taxonomical Characterization of Potential Isolate

**FIGURE 2:**
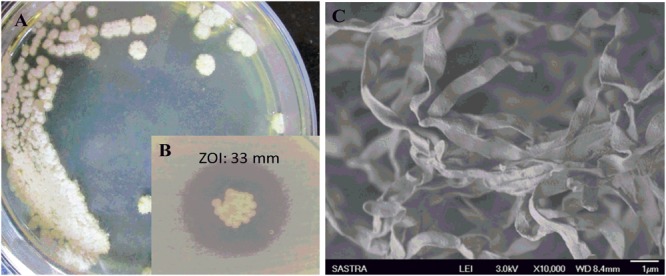
**(A)** Colony morphology of *Streptomyces* sp. ASK2 on ISP2 agar medium; **(B)** Antagonistic activity against *K. pneumoniae* by colony overlay assay; **(C)** Scanning electron micrograph of *Streptomyces* sp. ASK2 displaying branched ribbon like spores with smooth surface.

**FIGURE 3:**
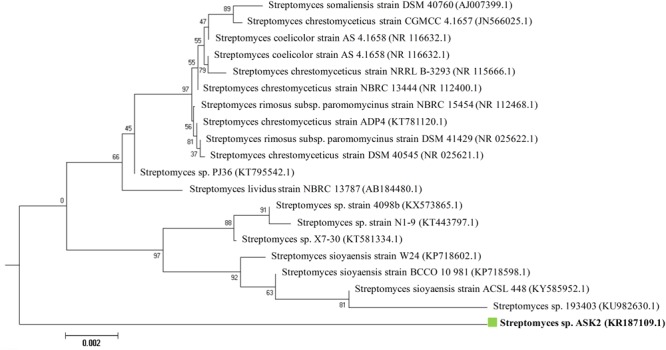
**Neighbor-joining phylogenetic tree based on 16S rRNA gene sequences showing the relationship between *Streptomyces* sp.** ASK2 and representatives of related species.

During the study, 51 morphologically different actinomycetes spp. were isolated from rhizosphere soil of five different medicinal plants. However, only one isolate was shown to have a potential activity against MDR *K. pneumoniae*. The isolate *Streptomyces* sp. ASK2 obtained from *Solanum trilobatum* soil sample displayed promising antagonistic activity with ZOI of 33 ± 0.5 mm by colony over lay assay (**Figure 2B**). Similarly the culture supernatant exhibited strongest activity against drug resistant *K. pneumoniae* and thus the strain ASK2 was subjected for taxonomical studies. The strain ASK2 was characterized by wrinkled, rough, irregular, dry and white aerial mycelia on ISP2 agar medium (**Figure 2A**). The scanning electron micrograph of ASK2 was found to have branched ribbon like spores with smooth surface (**Figure 2C**). The sequence (1053 base sequences) similarity search using BLAST tool reveals that ASK2 (Gen Bank Accession Number: KR187109) belongs to a distinct phyletic line in *Streptomyces* sp. The isolate was closely related to the type strain of *S. rimosus* subsp. paromomycinus strain NBRC 15454 sharing a homology of 97%. On the basis of cultural characteristics, spore structural properties, and 16S rRNA sequence, ASK2 was identified as a new strain belonging to *Streptomyces* sp. (**Figure 3**). The evolutionary history was inferred using the Neighbor-Joining method (Saitou and Nei, 1987; Ser et al., 2016). The optimal tree with sum of branch length = 0.04392832 was shown. The percentage of replicate trees in which the associated taxa clustered together in the bootstrap test (1000 replicates) were shown next to the branches (Felsenstein, 1985). The tree was drawn to scale, with branch lengths in the same units as those of the evolutionary distances used to infer the phylogenetic tree. The evolutionary distances were computed using the Kimura 2-parameter method (Kimura, 1980) and were in the units of the number of base substitutions per site. The rate variation among sites was modeled with a gamma distribution (shape parameter = 10). The analysis involved nucleotide sequences of 20 *Streptomyces* sp. All ambiguous positions were removed for each sequence pair. There were a total of 1520 positions in the final dataset. Evolutionary analyses were conducted using MEGA5 (Tamura et al., 2011).

### Multistep Purification and Identification of Active Peak

To isolate the active component, the crude extract (3 g) was subjected to reverse phase HPLC using optimized experimental parameters. Among the 12 fractions collected, the 3rd fraction with a retention time of 4.57 min showed potential antibacterial activity using broth dilution method. The re-passage of the active peak over the same column under the same experimental conditions yielded three peaks, among which one prominent peak at a retention time of 4.4 min was found to have bioactivity against *K. pneumoniae*. The compound purity obtained at the second re-passage was 86%. Hence the active peak was subjected to third round of re-passage with 40% water and 60% methanol solvent system. The compound purity was increased to 93% (**Supplementary Image S1**). The compound showed the killing effect on standard NDM type *K. pneumoniae*. The pure compound obtained was white solids and are soluble in H_2_O, DMSO, methanol and insoluble in CDCl_3_ and petroleum ether.

**FIGURE 4:**
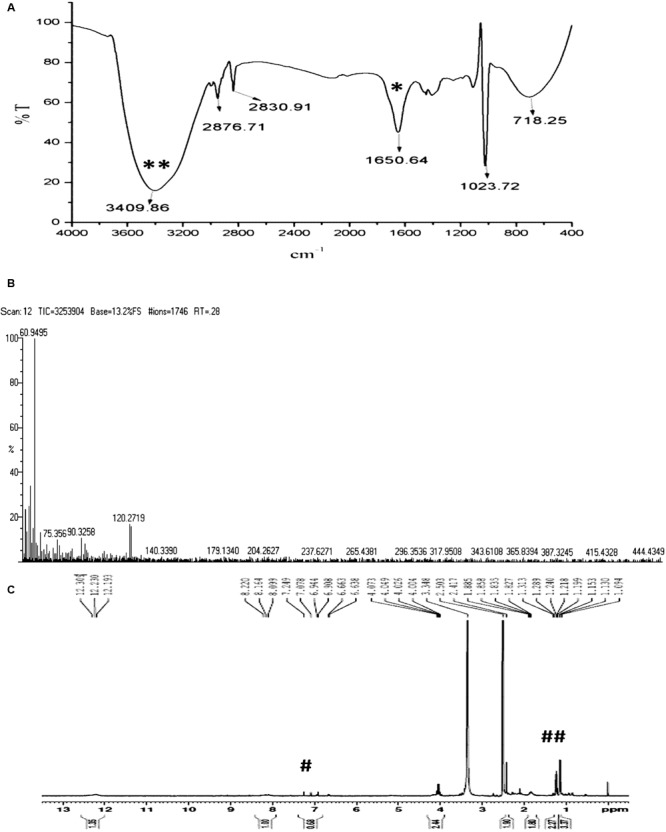
**Characterization of purified ASK2 bioactive molecule from *Streptomyces* sp.** ASK2: **(A)** FT-IR Spectra: Presence of strong absorption troughs at 3400 cm^-1^ (**) and 1651 cm^-1^ (*) representing the presence of OH and C=O groups; **(B)** ESI-MS spectra; **(C)** ^1^H NMR spectra represents the presence of aromatic compounds (#) and presence of hydrogen in aliphatic carbon chain (##).

In order to verify whether the compound is similar to that produced by *S. rimosus*, the HPLC comparison of ASK2 and oxytetracycline were made. HPLC comparison of oxytetracycline and ASK2 does not appear to have similar separation profile. Moreover, the FT-IR spectrum of oxytetracycline and ASK2 were different. IR spectrum of ASK2 showed strong absorption troughs at 3400 and 1651 cm^-1^ that represents the presence of OH and C=O groups, respectively (**Figure 4A**). Whereas, primary amine is the main functional groups in oxytetracycline, the ASK2 did not show any absorption peaks for primary amine (two peaks at ≈3400 cm^-1^ and ≈3500 cm^-1^), (Ramasamy et al., 2014). ESI MS data reveals that the molecular weight of ASK2 is found to be 444.43 Da (**Figure 4B**), which was found to be differing from oxytetracycline (460.43 Da) (Singh et al., 2011). In addition the ^1^H NMR showed the triplet peaks at 6.944 to 7.249 ppm. It indicates the presence of hydrogen moiety in aromatic environment. Peaks in the range of 1.199 to 1.313 ppm indicate the presence of aliphatic nature of carbon chain (**Figure 4C**). Hence the bioactive compound isolated from *Streptomyces* sp. ASK2 is warrant to be a new compound, however, other NMR spectrometric methods are required to elucidate the complete structural details of the compound.

### Screening of Toxicity and Efficacy of ASK2 in Zebrafish Model

Prior to testing of compound, the MIC of ASK2 was estimated for the purified compound. 16 μg/mL was found to be the MIC. The agar plates were recorded for the absence of colonies at this concentration. And the OD_600_ was comparable with the control (Nutrient broth + compound) for final concentrations of 16 μg/mL and above.

### Optimization of Subclinical and Clinical Infection for Planktonic Cells

We examined the susceptibility of zebrafish to *K. pneumoniae*. The adult fish were injected intramuscularly with various infectious dose (10^4^–10^14^ cfu/ml) to induce subclinical and clinical infection in zebrafish. The survival rate of each infectious dose is given in **Table 2**. Upon infection with 10^14^ and 10^12^ cfu/ml, zebrafish readily became infected displaying 100% mortality within 24 and 120 h, respectively. Whereas, upon infection with less than 10^12^ cfu/ ml, zebrafish were active and no mortality was observed (**Table 2**). Even though the aquarium water was highly loaded with *K. pneumoniae*, cells less than 10^12^ cfu/ml does not lead to clinical symptoms, suggesting that intramuscular injection with optimum dosage of *K. pneumoniae* are crucial to enhance the intracellular invasion and spread. Further, the estimation of colony forming units from tissue homogenates of the infected fish of each group on UTI agar reveals the presence of large numbers of *K. pneumoniae* organisms. These results demonstrated that *K. pneumoniae* successfully infects zebrafish and cause clinical illness. *K. pneumoniae* is considered to be the most important histamine producing bacteria in fish (Kanki et al., 2002). It is also known to cause fin and tail disease in Rainbow trout and more reports on *Klebsiella* infection in different fishes are documented (Diana and Manjulatha, 2012).

**Table 2:** Optimization of infectious dose of *K. pneumoniae* in zebrafish (planktonic cells).

Dose of challenge	Type of infection	*Klebsiella* burden in muscle cultured on UTI agar medium	Survival rate (%)				
			24 hpi	48 hpi	72 hpi	96 hpi	120 hpi
10^4^ cfu/ml	Sub-clinical	1 ± 0.5 × 10^5^ cfu/ml	100	100	100	100	100
10^6^ cfu/ml	Sub-clinical	2 ± 0.4 × 10^6^ cfu/ml	100	100	100	100	100
10^8^ cfu/ml	Sub-clinical	2.6 ± 0.3 × 10^6^ cfu/ml	100	100	100	100	100
10^10^ cfu/ml	Sub-clinical	2.8 ± 0.3 × 10^6^ cfu/ml	100	100	100	100	100
10^12^ cfu/ml	Clinical	3.8 ± 0.6 × 10^6^ cfu/ml	90	50	35	30	0
10^14^ cfu/ml	Clinical	3.2 + 0.7 × 10^7^ cfu/ml	0	0	0	0	0

*Experiment was repeated thrice on three independent days, *n* = 5.*

### Virulence of *K. pneumoniae* Planktonic and Biofilm

The role of different phenotypic expression (planktonic and biofilm) of *K. pneumoniae* were examined in zebrafish free living and biofilm model. Adult zebrafish were challenged with different concentrations of *K. pneumoniae* in planktonic and biofilm state. Biofilm at the dose of 10^12^ cells, caused a significant clinical symptoms and 100% mortality within 120 h. The lower dose (<10^12^ cfu/ml) leads to sub-clinical infection alone. These observations were similar to that of planktonic cells. Furthermore, the colony counts of tissue homogenate were similar to that of planktonic cells. The results show no correlation between severity of infection and different phenotypes of *K. pneumoniae* in zebrafish planktonic and biofilm infection model (**Table 3**).

**Table 3:** Effect of biofilms of *K. pneumoniae* in zebrafish (Biofilm).

Bacterial inoculums	*Klebsiella* burden in muscle	Survival rate (%)				
		24 hpi	48 hpi	72 hpi	96 hpi	120 hpi
10^8^ cfu/ml	2.3 ± 0.5 × 10^6^ cfu/ml	100	100	100	100	100
10^10^ cfu/ml	2.8 ± 0.4 × 10^6^ cfu/ml	100	100	100	100	100
10^12^ cfu/ml	3.9 ± 0.7 × 10^6^ cfu/ml	85	50	40	35	0

*Experiment was repeated one time on three independent days, *n* = 5.*

### Toxicity Assessment by Fixed Dose Procedure

To assess the non-lethal toxicity, various concentrations of ASK2 were evaluated. Zebrafish challenged with different concentrations were observed for behavioral changes and mortality for upto 120 h. The compound was non-lethal upto 64 μg/ml concentration, whereas doses greater than 64 μg/ml was toxic, and 100% mortality were observed for 128 μg/ml (**Table 4**). However, to screen for liver toxicity, the low dose (24 μg/ml), effective dose (48 μg/ml) and high dose (128 μg/ml) of ASK2 were evaluated by performing MTT reduction assay, fluorescent microscopic technique, and histopathology.

**Table 4:** *In vivo* toxicity of different concentrations of compound (*n* = 5).

Concentration of compound (μg/ml)	Survival rate (%)				
	24 h	48 h	72 h	96 h	120 h
8	100	100	100	100	100
16	100	100	100	100	100
32	100	100	100	100	100
64	100	60	60	60	60
128	20	0	0	0	0

### Hepatotoxicity Assessment in *Ex Vivo* Culture of Zebrafish Liver

**FIGURE 5:**
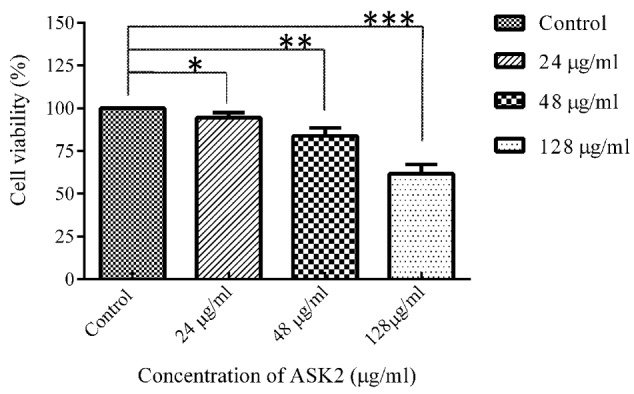
**MTT assay of zebrafish liver cells in *ex vivo* conditions.** Student’s *t*-test was performed to each treated group comparing with control. 24 μg/ml (*p = 0.0337), 48 μg/ml (**p = 0.0040), 128 μg/ml (***p = 0.0003).

**FIGURE 6:**
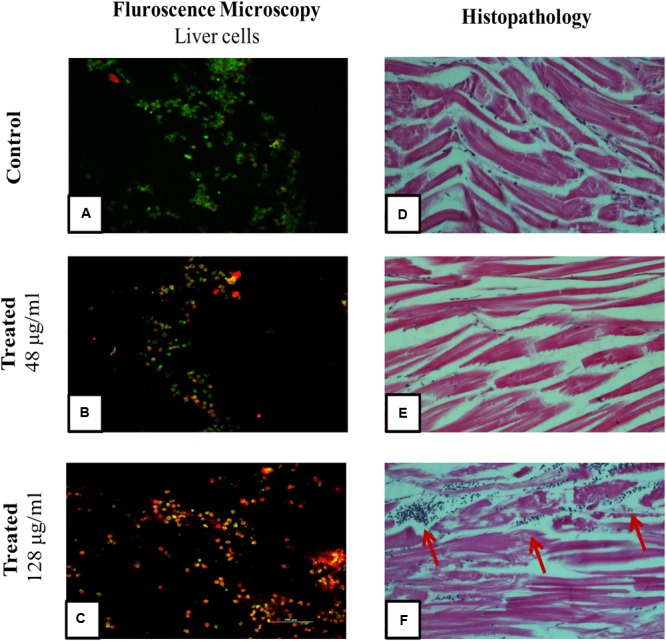
**Fluorescent Microscopic images of liver cells and Histopathology of zebrafish muscle after exposure to ASK2 compound: Liver cell viability by fluorescence microscopy, (A)** Control; **(B)** Treatment with 48 μg/ml ASK2; **(C)** Treatment with 128 μg/ml ; Muscle tissue histopathology; **(D)** Control; **(E)** Treatment with 48 μg/ml ASK2; **(F)** Treatment with 128 μg/ml (Arrows point to cellular infiltration in muscle, possibly owing to toxicity of the compound). However, no such changes were seen in Control and; **(E)** muscle tissue histopathology.

To evaluate cytotoxic activity of ASK2, the zebrafish were challenged with either low dose, effective dose or high dose of the compound. The treated and control groups were sacrificed after 7 h exposure and liver was obtained. The liver were carefully washed, trypsinized and grown in cell culture medium and incubated for 12 h. After the incubation period, the monolayer cultures were checked for cross contamination and light microscopic examination revealed the presence of adherent liver cells in microtitre plate. The cell viability by MTT assay displays that the compound induced hepatotoxicity in a concentration dependent manner (**Figure 5**). Zebrafish challenged with 24, 48, and 128 μg/ml compound shows 94, 83, and 61% relative cell viability, respectively (**Figure 5**). In addition, the florescent microscopic image depicts the toxicity by discriminating dead cells and viable cells. The effective dose (48 μg/ml) was found to have more viable cells (**Figure 6B**) similar to control (**Figure 6A**) compared to high dose (128 μg/ml) (**Figure 6C**). The statistical significance by Student’s *t*-test for 24 μg/ml was *p* = 0.0337, 48 μg/ml; *p* = 0.0040 and 128 μg/ml; *p* = 0.0003.

### Histopathology

A histopathological analysis of muscle after injection of the ASK2 compound was performed to check for toxicity of the compound. As can be seen from **Figure 6**, exposure of zebrafish to ASK2 compound at a concentration of 48 μg/ml (**Figure 6E**) did not produce any adverse change in the muscle, the site of injection of the compound. The muscle showed normal morphology as that observed with the control fish (**Figure 6D**). However, in the case of zebrafish that were exposed to the compound at a higher dose, 128 μg/ml (**Figure 6F**), there were focal points of cell infiltration into the muscle but the overall morphology of the muscle in this case also appeared to be normal. This suggests that the compound ASK2 is not toxic at the effective dose of 48 μg/ml, used to treat *Klebsiella* infection.

### Efficacy of Compound to Treat *Klebsiella* Infection

**FIGURE 7:**
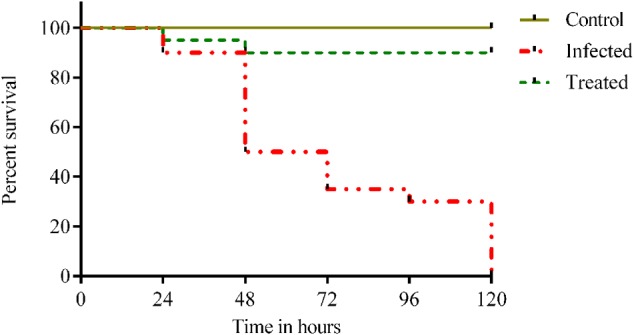
**Efficacy studies of ASK2 in *in vivo* conditions: Treated group was showing 90% survival up to 120 h, where as 100% mortality was observed within 120 h in untreated group compared to control group**.

Injection of 10^12^ cfu/ml of *Klebsiella* inoculum to the healthy adult fish showed clinical infection and 100% mortality within 120 h. Whereas, 90% of survival rate were observed for the treatment group after being treated with 48 μg/ml of ASK2 compound. Also, there was visible reduction in *Klebsiella* burden after 24 hpi in zebrafish treated with effective dose of ASK2 compound (**Figure 7**). The significant increase in survival rate at intermediate concentration clearly indicates that ASK2 exerts a potential therapeutic effect by controlling *K. pneumoniae* proliferation.

## Discussion

As the burden of MDR *K. pneumoniae* is growing rapidly, discovery of new drugs is an indispensable process to combat those emerging resistant pathogens (Bérdy, 2012). Actinomycetes are still the promising candidates for the discovery of novel antibiotics which are being isolated from wide range of natural habitats (Tiwari and Gupta, 2012). Exploring the untapped region for collection of rare and novel microbial species is challenging for the exploration of novel drugs (Cragg and Newman, 2013). However, we believe that potential antibiotics producers are still hidden in very familiar sources like soil, plants, marine plant and animals etc. Rhizosphere soil of medicinal plants are one such striking source of diverse microbial community (Köberl et al., 2013). In the present study, 51 different actinomycetes spp. were isolated from rhizosphere soil of five different medicinal plants. *Streptomyces* sp. ASK2 isolated from *Solanum trilobatum* was found to have a potential inhibitory activity against MDR *K. pneumoniae*. *Solanum trilobatum* is an important plant in medicine with wide applications including treatment of respiratory diseases, tuberculosis, cardiac and liver infection. It is also familiar for anti-inflammatory, anti-oxidant and immunostimulatory properties (Priya and Chellaram, 2014; Venugopal et al., 2014).

*Streptomyces rimosus* is well-known for the synthesis of oxytetracycline, possessing broad spectrum antibacterial activity and some bacterial strains have developed resistance (Chopra and Roberts, 2001). However, the usage of oxytetracycline for humans is prohibited as it is associated with adverse toxic effects. Hence it is now generally used to treat rickettsiae and mycoplasma infection in animals (Stuen and Longbottom, 2011).

The significance of toxicological screening are well recognized as an important step in drug development process, hence several zebrafish models and assays have been developed to study drug toxicology this decade (Bhusnure, 2015). In the current report, we have modeled *K. pneumoniae* infection in adult zebrafish, to study toxicity and efficacy of ASK2 isolated from *Streptomyces* sp. ASK2.

Since there are no reports on *K. pneumoniae* zebrafish infection model, we decided to optimize infectious dose, which are quite varied with strain, host susceptibility and even the route of transmission. Sub-clinical and clinical infections were established with infectious dose of 10^4^–10^10^ and 10^12^–10^14^ cfu/ml, respectively (**Table 2**). A similar kind of observation was recorded for *E. faecalis* in a dose-dependent manner in zebrafish larvae. In general, most of the studies with zebrafish embryo and early larvae used bacterial density of 10^3^–10^8^ cfu/ml to study host pathogen interactions and to predict drug safety. However, the infectious dose and pathogenicity are varied with pathogens belonging to same species of different strains. For instance, in *Galleria mellonella* infection model, Wand et al. (2013), demonstrated that different strains of *K. pneumoniae* showed different virulent expression. And another study revealed that different strain of same species can exhibit different degree of pathogenicity *in vivo* model (Lavender et al., 2004).

Though several zebrafish infectious models have been developed (Neely et al., 2002), the effect of drug to treat biofilm related infections are very limited using zebrafish (Chu et al., 2014). Infact, many studies have suggested that biofilm formation is an important phase in the infective process. Therefore, we investigated the significant role of planktonic and biofilm state of *K. pneumoniae* to cause infection. In our study, *K. pneumoniae* biofilm being formed *in vitro* were analyzed for its virulence in zebrafish. We observed a similar kind of pathological consequences in zebrafish planktonic and biofilm model. The infectious dose, severity of illness, survival rates and bacterial counts in muscle homogenates were similar for biofilm and planktonic zebrafish infection models (**Tables 2, 3**). One possible reason for similar pathological observation is the gene expression for biofilm formation on solid support is different from those required to establish *in vivo* (Lavender et al., 2004). Many studies have explored biofilm formation as a virulence phenotype in majority of the bacteria. For example, in a study with *Streptococcus suis* zebrafish model, *S. suis* biofilm had LD50 values greater than the LD50 values of planktonic cells, the virulence of biofilm cells were weaker than planktonic cells (Wang et al., 2011). However, in our case, the pathological consequences are similar for planktonic and biofilms. Our result suggests that biofilm state of the pathogens is not exclusively required for virulence expression. However, screening of biofilm establishment in zebrafish and validating with different strains of *K. pneumoniae* are essential to confirm our findings which is our lab’s future focus.

Once the infectious doses were optimized, we investigated the efficacy of ASK2 compound and toxicity in adult zebrafish, because drug induced liver injury is a major toxicological problem. The similarity of zebrafish liver functions and drug metabolism to that of humans, zebrafish has been used as good model for drug toxicity (Triebskorn et al., 2004; Zodrow et al., 2004; Devi et al., 2015; Ayaz Ahmed et al., 2016; Lowrence et al., 2016). The most commonly used drug induced hepatotoxicity assay in laboratory animals includes serum enzyme assay and histological assay (McGrath and Li, 2008). The present study assessed the liver injury by measuring MTT reduction to determine the cell viability for *ex vivo* culture of liver. There were no obvious changes in liver cell viability and metabolic activity, during *ex vivo* culture of liver from healthy adult zebrafish. The cytotoxic effect of ASK2 was observed in a dose dependent manner in zebrafish. The effective dose (48 μg/ml) of ASK2 displayed 83% liver cell viability, similarly no mortality were recorded during *in vivo* toxicity study. However, 61% liver damage within 6 h was recorded for treatment group with high dose (128 μg/ml), that is at eightfold of MIC and at the same point, 100% mortality were recorded within 24 h. The results of *ex vivo* culture of liver toxicity are highly co-related with *in vivo* toxicity and histopathology analysis, suggesting that *ex vivo* culture of zebrafish liver are a highly reliable and reproducible method for the assessment of drug induced toxicity. In an earlier study, zebrafish brain was similarly cultured *ex vivo* to study the effects of ethanol and acetaldehyde (Zenki et al., 2014).

The efficiency of drug to treat infected zebrafish showed that ASK2 compound was highly effective in killing and controlling *K. pneumoniae* proliferation. The obvious reduction of 10^12^ to 10^4^ cfu/ml bacterial burden was recorded with an improvement of 90% survival rate. The efficiency of the compound was highly comparable with untreated group which showed 100% mortality at 120 hpi (**Figure 7**). Hence, ASK2 compound isolated from *Streptomyces* sp. ASK2 was shown to be effective for the management of MDR *K. pneumoniae* infection in zebrafish model.

## Conclusion

The continuous use of similar kind of drugs for treatment and overuse of antibiotics in both human and livestock induces different drug resistance mechanisms in bacteria. Since there are no novel antibiotics in drug development in the present century for carbapenemase producing enterobacteriaceae, the existing antibiotics have re-emerged for current medications. Hence in response to address these issues the present research has come up with a potential anti-MDR *K. pneumoniae* molecule from *Streptomyces* sp. ASK2. The antibacterial compound ASK2 reported in the present study was shown to control MDR *K. pneumoniae* infection in zebrafish infection model with a significant improvement in fish survival rate. Furthermore, the *in vivo* toxicity, MTT assay of *ex vivo* liver culture, and histopathology results strongly support the non-toxic property of ASK2 at effective dose. These findings support the potentiality of ASK2 to treat MDR *K. pneumoniae* infection in zebrafish model. Further investigations on complete structural elucidation and extensive evaluation of chronic toxicity may lead to a novel drug candidate to combat the persistent MDR *K. pneumoniae*.

## Author Contributions

All authors listed, have made substantial, direct and intellectual contribution to the work, and approved it for publication.

## Conflict of Interest Statement

The authors declare that the research was conducted in the absence of any commercial or financial relationships that could be construed as a potential conflict of interest.

## Supplementary Material

IMAGE S1 **Isolation of bioactive molecule by Multi step reverse phase HPLC purification: (A)** Crude extract: *active peak at RT 4.57 min with 23% purity **(B)** Re-passage of *active peak eluted at RT 4.4 min with 86% purity; **(C)** Re-passage of *active peak eluted at RT 2.23 min with 94% purity.
